# CARE MANAGER PERCEPTIONS OF A CARE COORDINATION INTERVENTION IN THE ED FOR RESIDENTS IN ASSISTED LIVING COMMUNITIES

**DOI:** 10.1093/geroni/igae098.3819

**Published:** 2024-12-31

**Authors:** Grace Wittenberg, Peter Serina, Nichole Stetten, Ann Reddy, Ellen McCreedy

**Affiliations:** Brown University School of Public Health, Eau Claire, Wisconsin, United States; Brown University School of Public Health, Providence, Rhode Island, United States; Brown University, Brown University, Rhode Island, United States; Brown University School of Public Health, Providence, Rhode Island, United States; Veterans’ Administration, Providence, Rhode Island, United States

## Abstract

Of the 800,000 residents in US assisted living communities, nearly half are transferred to the Emergency Department (ED) each year, often without a support person or up-to-date medical information. This lack of information can lead to more testing in the ED, increased ED length of stay, and unnecessary hospitalizations. To address this problem, Bluestone Physician Services, providing care to ALC residents in Florida, Minnesota, and Wisconsin, developed the ED Early Response Program for patients in its accountable care organization. In the program, a complex care manager (CCM) is notified when one of Bluestone’s ALC patients registers at an ED. Bluestone staff then provide standardized, clinical information to the ED via phone and fax. To analyze the feasibility and acceptability of the program, we conducted semi-structured interviews with 12 CCMs involved in the program. Using directed content analysis, we identified five overarching themes: populations most likely to benefit from the program (e.g. people living with dementia, hospice), key components of the program, program strengths, areas of opportunity, and impact on patient care. CCMs described that the program positively impacted care transitions to the ED by providing added clinical information and fostering interprofessional communication. However, they also noted areas for improvement, including enhancing ED provider receptivity by increasing awareness of the program in the ED. Overall, by using CCM insights gained during implementation, this program can be used as a model to address key communication gaps for ALC residents during transitions of care, including ED visits.

